# Do Age-related Differences in the Incidence of Mumps Deafness Reflect a True Difference or a Misclassification of Mumps Deafness?

**DOI:** 10.2188/jea.JE20210002

**Published:** 2022-01-05

**Authors:** Takashi Fujiwara, Yohei Maeda

**Affiliations:** 1 Department of Public Health Research, Kurashiki Clinical Research Institute, Okayama, Japan; 2 Department of Otorhinolaryngology–Head and Neck Surgery, Osaka University Graduate School of Medicine, Osaka, Japan

**To the Editor:**

We read the article by Takagi et al with great interest and appreciate their effort to assess the incidence of mumps deafness in mumps cases.^1^ In a claim-based database study, the authors found that the incidence of mumps deafness was 8.4 times higher in adolescents and adults than in children (73.6 per 10,000 patients for adolescents and adults and 6.6 for children). However, the estimate might be invalid because of the definitions of mumps and mumps deafness used.

The authors used the “B26 code from the International Statistical Classification of Diseases and Related Health Problems, 10^th^ Revision (ICD-10)” to identify mumps cases. The validity of the claim diagnosis code has not been evaluated in Japan,^2^ and we are concerned regarding the underestimation of mumps due to mumps patients seen in a clinical setting who are not given a B26 code. The treatment of mumps is analgesics and cooling the affected area, and there is no specific medication for mumps. When a physician sees a mumps patient, the physician needs to give the patient an analgesia-related ICD-10 code (eg, headache or lower ear pain) for insurance, but does not need to give the ICD-10 B26 code. In Japan, sentinel surveillance is conducted, and there are 0.431 to 1.356 million suspected mumps cases annually.^3^ The Japanese Medical Data Center (JMDC) database is based on employment-based health insurance claims, and it mainly covers the population younger than 65 years.^4^ The database contained 5.5 million subjects as of June 2018,^1^ and accounted for about 6.1% of the total population of Japan younger than 65 years.^5^ Based on a sentinel surveillance rate of 6%, there should be 26,860 to 81,360 cases of pediatric mumps annually. However, Takagi et al identified only 62,551 pediatric mumps cases during their 12-year study.

The authors also used an “acute sensorineural deafness and mumps antibody test in the same month” as the third definition of mumps deafness. Claim-based databases, including the JMDC database, do not have the results of laboratory tests. If a patient has acute sensorineural deafness and has the mumps antibody test to rule out mumps deafness, the patient is classified as “mumps deaf” in the study. Indeed, otolaryngologists occasionally perform the mumps antibody test on acute sensorineural deaf patients without swelling of the parotid glands. Since one of the main cause of pediatric acute sensorineural deafness is mumps deafness, the definition would be acceptable for children. However, mumps deafness is an unusual cause of adult acute sensorineural hearing loss, with the major cause being sudden hearing loss. The incidence of sudden hearing loss increases with age and is about eight times higher in adolescents and adults than in children,^6^ and the age-related difference in sudden hearing loss is similar to that observed in Takagi et al’s study.

Therefore, a major limitation of the study by Takagi et al is that the lack of a validation study of the outcome definition might lead to imprecise case definitions for mumps and mumps deafness. Consequently, the age-related difference they observed would not be the true difference in the incidence of mumps deafness.
